# Peritoneal patch in portal vein reconstruction: Evaluating graft material outcomes in hepatopancreatobiliary and liver transplant surgery - A Systematic Review

**DOI:** 10.12669/pjms.41.4.10465

**Published:** 2025-04

**Authors:** Saira Imtiaz, Ibtissam Bin Khalid, Zulqarnain Hyidar, Arslan Saleem Chughtai, Muhammad Yasir Khan, Irfan Ahmed

**Affiliations:** 1 Saira Imtiaz, Pakistan Kidney and Liver Institute and Research Center, Lahore, Pakistan; 2 Ibtissam Bin Khalid, Shaukat Khanum Memorial Cancer Hospital & Research Center, Lahore, Pakistan; 3 Zulqarnain Hyidar, Shaukat Khanum Memorial Cancer Hospital & Research Center, Lahore, Pakistan; 4 Arslan Saleem Chughtai, Pakistan Kidney and Liver Institute and Research Center, Lahore, Pakistan; 5 Muhammad Yasir Khan, Pakistan Kidney and Liver Institute and Research Center Lahore, Pakistan; 6 Irfan Ahmed, Pakistan Kidney and Liver Institute and Research Center, Lahore, Pakistan

**Keywords:** Peritoneal patch, Portal vein reconstruction, Graft material, Systematic review, Safety, Effectiveness

## Abstract

**Background & Objective:**

In hepatopancreatobiliary and liver transplant surgeries, portal vein repair is often needed. Peritoneal patches (PPs) might provide an advantage over synthetic grafts for portal vein reconstruction. The objective of this systematic review was to compare clinical outcomes, safety, and effectiveness of PPs versus conventional graft materials.

**Methods:**

A comprehensive search of multiple databases such as PubMed, CINAHL EMBASE, SpringerLink Pakmedinet, and Google Scholar was conducted for studies focusing on portal vein repair using PPs. Eligible studies were case reports, case series, and retrospective studies from January 2000 to December 2024. Due to the heterogeneity of study designs and outcomes, a qualitative synthesis approach was applied.

**Results:**

Six studies were included. The studies reported a variety of surgical techniques and outcomes. Autologous peritoneal interposition grafts and PPs resulted in effective intraoperative portal flow and patency. Some studies, despite generally positive outcomes, reported complications such as thrombosis and significant postoperative events. A rigorous risk of bias assessment was not performed due to the limitations of the study design.

**Conclusion:**

PPs may potentially be an alternative for portal vein reconstruction in HPB and LTx surgeries that offer benefits such as availability, adaptability, and lower immunogenic or thrombotic risks. These results should be confirmed with further research in the forms of prospective and comparative studies.

## INTRODUCTION

Portal vein reconstruction is a very important component of hepatopancreatobiliary and liver transplant surgeries. Vascular integrity will determine surgical success, especially because it keeps blood flow to the liver.^1^ The portal vein carries nutrient-rich blood from the gastrointestinal tract into the liver and plays an essential role in hepatic circulation.^2^-^4^

Vascular reconstruction employs various materials, including autologous veins, prosthetic materials, and peritoneal grafts.^5^ The use of autologous veins is preferred due to their biocompatibility and low thrombogenicity, but they are technically challenging and require additional incisions.^3^ Prosthetic materials are easily available but pose a higher risk of infection.^6^ The peritoneum presents a promising alternative; it provides a vascular graft of desired length and diameter with reduced preparation time and technical demands.^6^,^7^ In cirrhotic patients, the risk of infection may be increased, especially in those with recurrent spontaneous bacterial peritonitis.^7^

The choice of graft material has a significant influence on surgical outcomes, patient morbidity, and graft survival. 5,6 New advances in surgical techniques and the advent of synthetic and biological grafts have expanded options for portal vein reconstruction.^7^-^9^ Still, the literature is limited to case reports and small cohorts that mainly involve liver resections.

This systematic review aimed to evaluate the efficacy and outcomes of peritoneal patch grafts for portal vein reconstruction in HPB and LTx surgeries. By synthesizing available evidence, the review seeks to provide insights into the advantages, limitations, and potential role of peritoneal patches as a viable option for vascular reconstruction in these complex procedures.

## METHODS

In this systematic review such literature/ studies has been included that were focused on published original articles, observational and experimental trials, regarding portal vein reconstruction using Peritoneal Patches (PP). Thesis/ posters and unpublished online available literature were excluded from the study. Due to the limited availability of relevant literature on this subject, we considered various study designs, including case reports, case series, and respective studies.

### Information sources:

**Fig.1:**
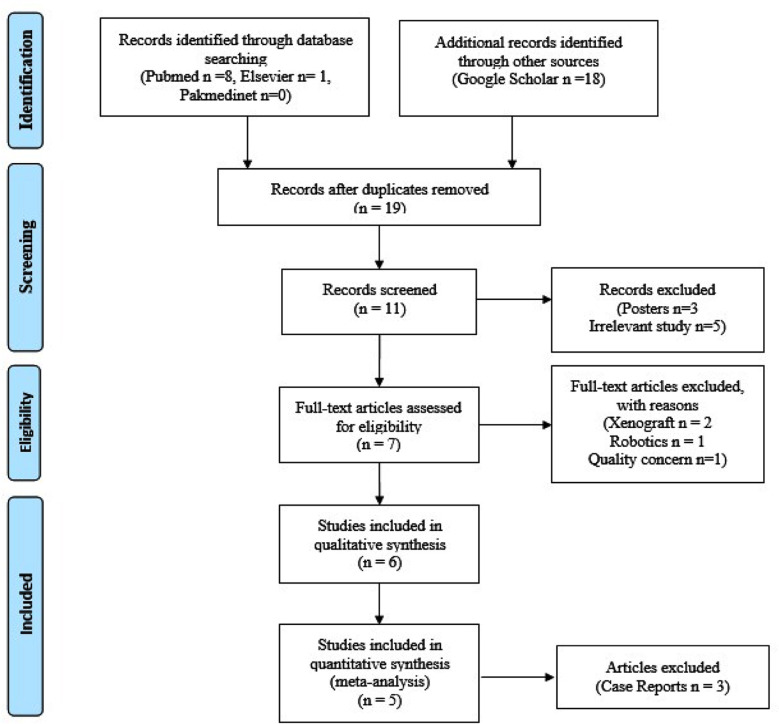
PRISMA flow diagram for the study selection.

We conducted a comprehensive literature search using multiple databases and search engines. Specifically, we searched for the following sources:

- *PubMed*: We retrieved eight relevant studies, with a focus on those published within the years 2000-2024, resulting in two studies meeting the eligibility criteria.
- *CINAHL:* No relevant study identified i.e. meeting the selection criteria.
- *Embase:* One study was found.
- *SpringerLink:* No study was found.
- *Pakmedinet:* Search did not yield any relevant studies.
- *Google Scholar:* We searched within the years 2000-2024, initially yielding 122,000 results. After applying our search criteria, we narrowed the results to 17,400. Further refinement led to 5,480 relevant results (Fig.1).

**Fig.2:**
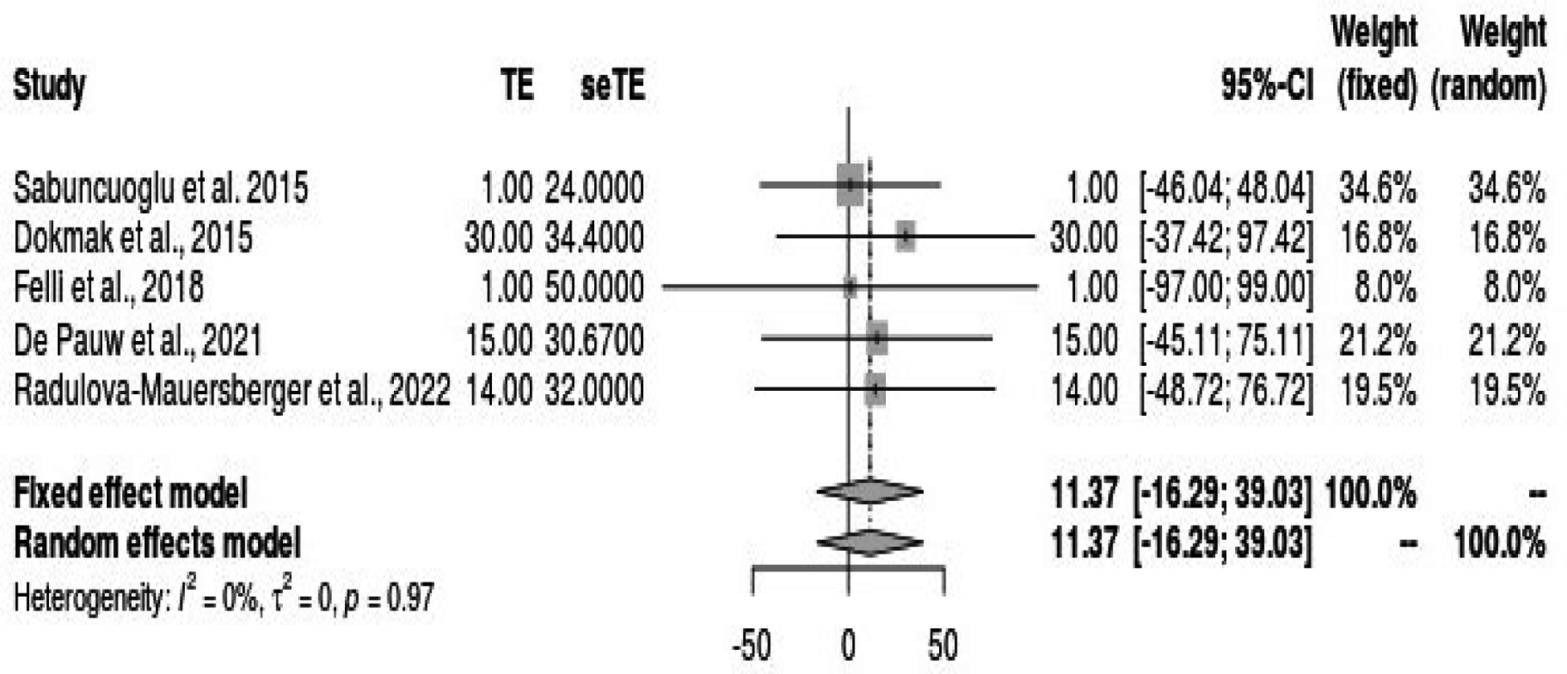
Peritoneal patch length (mm) use.

### Search strategy:

We utilized medical subject heading (MeSH) terms to identify relevant keywords for our search strategy. The MeSH terms included “Peritoneal patch,” “Portal vein,” and “Repair/reconstruction.” These terms were combined using “and” and “or” Boolean operators to ensure a focused search on portal vein reconstruction using PPs.

### Selection process:

Identified studies were screened by two independent reviewers for relevance to the research question and inclusion criteria. Some studies were excluded due to being unavailable in full text. The scarcity of relevant studies underscores the novelty and niche nature of the topic, necessitating a scoping systematic review approach rather than a meta-analysis.

### Data collection process and data items:

Data were extracted from the selected studies, focusing on pertinent outcomes related to portal vein reconstruction using PPs. Key data items including, Study Design, flow rate of Portal Vein Reconstruction, incidence of thrombosis, postoperative complications, length of hospital stay (LOS), and surgical outcome.

### Study risk of bias assessment:

Given the retrospective nature of the included studies, along with case reports and animal studies, a formal risk of bias assessment using standard tools such as the Cochrane Risk of Bias tool or the Newcastle-Ottawa Scale was not feasible. However, we critically evaluated the methodological quality and potential biases inherent in each study during data interpretation and synthesis. Factors such as study design, sample size, confounding variables, and potential sources of bias were considered in assessing the overall quality of evidence.

### Effect measures:

While specific effect measures were not predefined in this study, the following variables were identified for systematic review: study design, frequency of patients, age distribution, Gender distribution, type of graft used, flow rate of portal vein reconstruction, incidence of thrombosis, postoperative complications, length of hospital stay (LOS), surgical outcome. These variables were systematically collected and analyzed to evaluate various aspects of portal vein reconstruction using PPs. Analysis was not possible as data to assess surgical success rates, postoperative complications, and long-term outcomes were not available due to the heterogeneity of the study designs.

### Synthesis methods:

Data from the included studies were synthesized using a narrative synthesis approach. This involved summarizing findings from individual studies, identifying common themes, and discussing patterns across studies. Due to the heterogeneity of study designs and outcomes, meta-analysis was not feasible. Instead, qualitative synthesis methods were employed to provide a comprehensive overview of the evidence on portal vein reconstruction using PPs.

### Reporting bias assessment:

While efforts were made to minimize reporting bias by conducting a comprehensive literature search across multiple databases and search engines, no formal assessment of reporting bias was conducted. However, the potential for publication bias and selective reporting of outcomes was acknowledged and considered during data interpretation and synthesis.

### Certainty assessment:

A formal assessment of certainty or quality of evidence using tools such as the GRADE approach was not conducted due to the limited availability of high-quality studies, particularly randomised controlled trials. However, the strength of evidence from the included studies was considered when drawing conclusions and making recommendations. The limitations of the evidence base were also acknowledged and discussed within the context of systematic review.

## RESULTS

The included studies exhibited varying study designs and characteristics. Commonly reported variables across the studies included study design, number of participants, age distribution, gender distribution, type of graft used, low rate of portal vein reconstruction, incidence of thrombosis, postoperative complications, length of hospital stay (LOS), and surgical outcome. This case report by Marino et al. (2023) described a single patient undergoing total portal vein replacement using autologous parietal peritoneum grafts during Whipple’s procedure for extrahepatic cholangiocarcinoma. Adequate portal flow and patency were confirmed intraoperatively.^10^ De Pauw et al. (2023) conducted a retrospective study involving 15 patients undergoing PP reconstruction during pancreaticoduodenectomy. The study reported a thrombosis rate of 6.7% on day seven and a 40% incidence of major complications.^11^ Felli et al. case report presented the use of an autologous peritoneal patch for partial portal vein resection during left hepatectomy. A 46-year-old male with hepatocellular carcinoma and portal vein thrombosis who required partial resection of the portal vein and segments II and III of the liver was treated. Reconstruction of the portal vein was achieved using an autologous peritoneal patch harvested from the diaphragm. There was no requirement for extensive mesenteric root mobilization or prosthetic graft insertion. Postoperative recovery was uneventful, and the patient was discharged on the 7th postoperative day. Pathological examination revealed hepatocellular carcinoma (pT4), and there was no evidence of thrombosis or other vascular complications after the surgery.^12^

Radulova-Mauersberger et al. (2022) performed a retrospective study of 14 patients who underwent PPs reconstruction for the vena cava inferior and portal vein. The study reported a high success rate (97%) but noted clinically relevant complications in 24% of patients.^13^ The study by Dokmak et al.^8^ (2015) involved 30 patients undergoing venous reconstruction using autologous peritoneum in hepatopancreatobiliary surgery. The mean length of lateral patches was 22 mm, and tubular substitutes measured 30-35 mm. They reported a 90% rate of no or mild stenosis, with low complication rates (Clavien-Dindo grades I-III) and no mortality within a median postoperative period of 16 days.^13^ Sabuncuoglu et al. case report focused on the use of an autologous peritoneal graft to repair a portal vein injury caused by blunt thoracoabdominal trauma.

The subject was a 28 years old male who underwent portal vein reconstruction after severe trauma. The surgical team used an autologous peritoneal graft for the portal vein reconstruction to restore blood flow. Postoperatively, the hepatic artery was ligated, and external biliary drainage was placed. Unfortunately, the patient expired 12 hours after the surgery. Despite this, postmortem findings indicated that there were no signs of thrombosis, ischemia, or congestion in the liver or bowel, and portal vein blood flow had been successfully restored^14^ (Table-I).

**Table-I:** Results of individual studies.

Title	1^st^ Author	Study design	n	Age	Gender	Graft	Flow rate	Thrombosis	Complication	LOS	Outcome
Parietal Peritoneum as an Autologous Substitute for Venous Reconstruction in Hepatopancreatobiliary Surgery	Dokmak et al., 2015 8	Clinical trial	30	57(41-38)	19 Male, 11 Female	Peritoneum of the Diaphragm mean length of lateral patch was 22 mm (15–70) and the tubular autologous substitute was 30 and 35 mm	No or mild stenosis 27(90%)	1(3%)	Clavien-Dindo grade I was observed in 2 (7%), II in 8 (27%), and III in 4 (13%) patients	16(10-48) days	No mortality
Total portal vein replacement with peritoneal interposition graft during Whipple’s procedure for extrahepatic cholangiocarcinoma: a technical report	(Marino et al., 2023) 10	Case Report	1	67	Female	A 4×3 cm section of diaphragmatic Autologous Parietal Peritoneum Grafts (APG)	Adequate portal flow and patency were proven using intraoperative Doppler ultrasound	N/A	N/A	16 days	N/A
Peritoneal patch in vascular reconstruction during pancreaticoduodenectomy for pancreatic cancer: a single Centre experience	(De Pauw et al., 2023) 11	Retrospective	15	67 (63-71) *	10 Males 5 Females	Peritoneum Patch Venous reconstruction was performed using a PP sewn with continuous suture of non-resorbable monofilament (5/0).	N/A	1/15(6.7%) on day 7	Clavien-Dindo grade III and IV) were recorded in 6/15(40%) patients.	12 days (9.5–14.5). 2/15 patients (13.3%) readmitted within 12 days	A median follow-up of 5.7 months (5.1–7.7), all patients were alive and 2/15 (13.3%) developed disease recurrence with liver metastasis after 3 and 9 months respectively
Autologous Peritoneal Patch for Partial Portal Vein Resection During Left Hepatectomy	Felli et al., 2018 12	Case report	1	46	Male	Autologous peritoneal patch	Restored	No	None	7 days	Uncomplicated recovery, discharged on 7th day, pathology confirmed pT4 hepatocellular carcinoma
How we do it—the use of Peritoneal patch for reconstruction of vena cava inferior and portal vein in hepatopancreatobiliary surgery	(Radulova-Mauersberger et al., 2022)^13^	Retrospective	14	60 (53-71) *	N/A	Parietal peritoneum patches size varied between 150 and 3600 mm^2^	Obstruction in 2/17(12%)	None	Treatment 413.7 min (IQR: 311.5–511) and median blood loss was 1205.8 ml (IQR: 650–1650)	15(IQR 9.5-38) days (range 7-131 days)	Clavien-Dindo grade ≥3b need of intervention occurred in four (24%) of the patients
Using autologous peritoneal graft for portal vein injury due to blunt trauma	Sabuncuoglu et al. 2015^14^	Case report	1	28	Male	Peritoneal graft was used for the portal vein reconstruction; size was 6 cm in length and 4 cm in width.	Restored	No evidence of portal venous thrombus or bleeding was revealed	Hepatic artery ligation, external biliary drainage	Not applicable, as the patient passed away 12 hours post-surgery.	Patient expired 12 hours after the operation due to unidentified causes; postmortem examination showed no signs of ischemia or congestion in the liver or bowel.

* Median (IQR) age, N/A; not available, LOS; length of stay in hospital.

### Risk of Bias in Studies:

While no formal risk of bias (ROB) assessment was conducted, the methodological quality and potential biases of each study were considered during data interpretation. The diverse study designs and limited sample sizes were acknowledged as potential sources of bias. A careful analysis and synthesis of the available data was done to reduce bias.

### Results of Syntheses:

The synthesis’s findings provided crucial new information on how PPs are used in the included studies’ portal vein repair. Because of the varied nature of the data and research designs, quantitative synthesis or meta-analysis was not possible; nevertheless, qualitative synthesis offered insightful information about the experiences and outcomes documented in the literature. The qualitative synthesis brought to light the following important conclusions: PPs have been effectively employed in several surgical operations, such as pancreaticoduodenectomy, repair of the inferior vena cava, and vena cava and portal veins. PPs have proven to be effective as vascular replacements in several situations when adequate portal flow and patency were confirmed intraoperatively. However, several studies revealed substantial postoperative problems, including thrombosis, which highlights the need for meticulous surgical technique and patient selection. The qualitative synthesis offered insightful information on the viability and results of using PPs in portal vein reconstruction, even despite the limitations of the included studies, which included small sample numbers and retrospective designs.

### Reporting Biases:

Even if efforts were made to lessen reporting biases by conducting a thorough literature search and including a wide range of studies, publishing bias must still be noted. Only the published articles were considered in this systematic review, excluding the rest, which are not easily accessible. There is some impact of interpreting the selected results from the studies we included even then; we tried our best to address it by reporting and assessing as transparently as possible the study’s Risk of bias and quality.

### Certainty of Evidence:

The evidence’s overall quality was limited due to the frequency of case reports, retrospective research and only clinical trials. The safety and efficacy of PPs in portal vein repair were therefore generally viewed with low confidence. Notwithstanding these shortcomings, the systematic review was successful in pointing up significant gaps in the literature and shedding light on topics that need further research.

## DISCUSSION

### Interpretation of Findings:

The use of autologous peritoneal interposition graft (APG) in pancreatic-tendonectomy for extrahepatic cholangiocarcinoma by Marino et al., 2023, highlights the effectiveness of this approach in accomplishing radical tumour excision by ensuring complete oncologic clearance and avoiding positive margins (R1) at final pathology.^10^ PP was utilised by De Pauw et al., 2023, as a safe and practical venous replacement after pancreaticoduodenectomy (PD) with vascular reconstruction (VR), with an acceptable rate of postoperative morbidity. But a decline in venous patency after 12 weeks highlights the need for more research on anticoagulation treatment and casts doubt on the long-term efficacy of PP. 11

Felli et al. demonstrated the effectiveness of using an autologous peritoneal patch for portal vein reconstruction during planned liver resections in patients with hepatocellular carcinoma. The procedure allowed for successful resection without the need for complex vascular reconstruction techniques, such as mesenteric root mobilization or prosthetic graft placement. This positive outcome may be presumed to give an impression that peritoneal patches could be a simple and more efficient, less invasive alternatives to vascular reconstructions in elective liver surgeries. With no complications such as thrombosis or anastomotic failure, the technique’s success allows its use in patients undergoing partial portal vein resections.^12^

During hepatopancreatobiliary (HPB) surgery, reconstruction of the inferior vena cava (IVC) and portal vein (PV) with parietal PPs is a viable and efficient substitute for alloplastic or xenogenous grafts, with a low risk of perioperative vascular complications (Radulova-Mauersberger et al., 2022).^13^ The utilization of parietal peritoneum (PP) as an autologous substitute for venous reconstruction in hepatopancreatobiliary (HPB) surgery presents a promising avenue for addressing the challenges associated with vascular resections in a study by Dokmak et al., the authors explored the feasibility and efficacy of PP grafts in complex liver and pancreatic resections requiring venous resection. The results thus point towards the fact that PP grafts may be effective lateral patches or tubular substitutes for MPV, VC, and hepatic vein defects and show excellent outcomes in terms of vascular patency and postoperative morbidity.

The high prevalence of histological vascular invasion among cases underscores the oncological importance of achieving R0 resections with sufficient margins. Despite the technical complexity of HPB surgeries and the high prevalence of malignant tumors requiring vascular resections, the use of PP grafts facilitated successful venous reconstruction without compromising oncological principles. Notably, the absence of PP-related or hemorrhagic complications emphasizes the safety profile of this autologous substitute, offering reassurance to surgeons encountering unforeseen vascular resections during HPB procedures.^13^

Pancreaticoduodenectomy with en bloc resection of the superior mesenteric vein (SMV) lateral wall addresses challenging periampullary tumor invasions into portomesenteric veins. Sabuncuoglu et al. highlighted the viability of using an autologous peritoneal graft for portal vein reconstruction after traumatic injury. Despite the patient’s unfortunate outcome, the restoration of portal blood flow and the absence of thrombotic or ischemic complications indicate that peritoneal grafts may be a viable option in emergency trauma situations. The postoperative death of the patient may be attributed to other trauma-related factors, not directly linked to the vascular reconstruction. The technique could be an alternative in trauma surgery, where synthetic or other autologous grafts are unavailable or unsuitable, although more research is needed to confirm its broader efficacy.^14^

### Synthesis of Evidence:

Sometimes the primary suturing is inept to repair in treating the vascular injuries, for that, autologous peritoneal grafting is the best choice. It is also safe as it needs no more incisions and is also a better option against prosthetic material. For optimal outcomes, the collaborative multidisciplinary approach was highlighted in complex HPB cancers which also include shielding pancreatic tissue plus vascular reconstruction about peritoneal grafting. Felli et al. case report explored the use of an autologous peritoneal patch during elective liver surgery for hepatocellular carcinoma with portal vein involvement. The grafting procedure was successful in restoring portal vein flow and preventing thrombosis, with the patient having an uncomplicated recovery and being discharged on the seventh day post-operation. This suggests that the peritoneal patch is an effective, safe, and readily available alternative for vascular reconstruction in planned hepatectomy procedures. In elective surgical settings, the autologous peritoneal patch provides a safe and practical alternative for portal vein reconstruction, offering benefits such as reduced operative complexity and favorable outcomes, including restored flow and minimal complications.^12^

The study by Dokmak et al. supports the efficacy of autologous PP grafts in HPB surgery, matching or surpassing other substitutes in vascular patency and oncological outcomes. PP grafts offer versatility, rapid availability, and low complication rates, making them preferred in emergent vascular resections. Integrating evidence from this and prior studies highlights PP grafts’ acceptance and role in expanding vascular reconstruction options in HPB surgery, despite study variations. Their consistent favorable results point to their promise as a potential substitute for venous reconstruction, which further expands the surgical armamentarium in this field.^13^ Case report by Sabuncuoglu et al. discusses a trauma case where an autologous peritoneal graft was used for portal vein repair after blunt thoracoabdominal injury. The experiment showed that grafts from the peritoneum are very effective at re-establishing blood flow without promoting thrombosis or ischemia. Yet the patient died 12 hours later, an example of the multifactorial nature of trauma care and how other variables might influence what otherwise was technically successful vascular reconstruction. The emergency use of autologous peritoneal grafts could be feasible in situations in which synthetic material access or the use of other autologous veins is not possible. But long-term survival is warranted to continue exploring those cases.^14^

Homologous parietal peritoneum (HPP) and parietal peritoneum patches (PPPs) research offers a lot of information on graft histology, long-term patency, and remodeling, emphasizing the need for continuous monitoring of graft performance. Studies stress the significance of achieving R0 resection in aggressive solid pseudopapillary epithelial neoplasms (SPEN) and recommend vascular or multi-visceral excision when necessary. The necessity for portal vein restoration in pancreatic surgery cases is noted, with lateral or segmental grafts being required in a subset of patients. Various alternatives to vascular reconstruction exist, including autologous veins, polytetrafluoroethylene (PTFE) grafts, and cryopreserved veins, with peritoneal grafts emerging as a safe and effective option, particularly in unplanned lateral wall vascular reconstruction.^15^-^18^

Peritoneal grafts offer adaptability and simplicity for vascular abnormalities,^19^ confirmed by studies demonstrating their viability, safety, and cost-effectiveness, even in patients experiencing postoperative pancreatic leakage. These findings encourage surgeons to consider peritoneal grafts as a viable option for vascular reconstruction, particularly when oncologically indicated,^16^,^18^ thereby reducing surgeon reluctance and improving patient outcomes. The simplicity of the harvesting technique, along with no reported major complications related to the graft, underscores the potential of peritoneal grafts in future vascular reconstructions.^17^ Yoon et al. (2021) presented an animal study investigating the feasibility of using homologous parietal peritoneum as a vascular substitute. However, no patient data was provided as this was an animal study. Using homologous parietal peritoneum (HPP) as a vascular substitute for venous reconstruction in abdominal surgery shows promising results in terms of vascular patency but concerns regarding long-term patency and graft stability warrant further investigation. The inner diameters of the anastomotic sites were 6.23 ± 0.18, 5.64 ± 0.16, and 2.34 ± 0.21 mm in the 7-day, 14-day, and 28-day groups, respectively. The midpoint inner diameters of the homologous parietal peritoneum grafts were 624 ± 0.46, 5.74 ± 0.26, and 2.14 ± 0.28 mm in each group, respectively. Endothelial cell proliferation on the homologous parietal peritoneum graft surfaces in all groups was based on the histological findings from the first group.^20^ In a case report by Har et al. detailing the successful repair of an inadvertently cut donor inferior vena cava during living donor liver transplantation using parietal PPs adds significant clinical relevance. This case highlights the practical application and efficacy of PPs in addressing vascular complications, reinforcing the conclusions drawn in our systematic review regarding the utility of autologous peritoneum grafts in hepato-pancreato-biliary surgery.^21^

### Implications of Findings:

The results of the study imply that while assessing the use of autologous peritoneal grafts and PPs for vascular reconstruction as an alternative in the treatment of challenging HPB malignancies, surgeons should take long-term outcomes and patient-specific factors into account. These new techniques could result in better patient outcomes and fewer surgical complications compared to traditional approaches. However, further research is needed to establish long-term safety, robustness, and efficacy of these techniques. For the proper management of complex HPB malignancies, interdisciplinary teamwork and standardized techniques are crucial.

### Limitations

The small sample sizes and case reports included in the review limit its generalizability. Bias is introduced by variations in patient demographics and surgical techniques. To address these limitations and provide strong evidence, prospective, multicenter studies with bigger cohorts are required to demonstrate safety and effectiveness.

## CONCLUSION

Novel vascular reconstruction methods like homologous parietal peritoneum and autologous peritoneal grafts can be promising in complex HPB procedures.

### Future research directions:

Further trials with a large number of participants, long-term follow-up (for durability, patency, and functional outcomes), and comparison with other grafts should be performed in cases of complex HPB cancers to determine the most effective approaches for specific patient groups and clinical situations.

### Author’s Contribution:

**IA, IBK, ZH and MYK:** Conception and design. **SI, ASC, IBK and ZH:** Data acquisition.

**ASC and SI:** Data analysis and interpretation. **MYK, IA, IBK, ZH and SI:** Drafting and critical revision of the manuscript for important intellectual content.

All authors have approved the final version and are accountable for the integrity of the study.

## Availability of Data, Code, and Other Materials:

It will be made available upon request, as well as results, counting the full report plus supplemental materials, would be accessible publicly, via an open-access platform.

## References

[1] Khalil A, Quaglia A, Gélat P, Saffari N, Rashidi H, Davidson B (2022). New developments and challenges in liver transplantation. J Clin Med.

[2] Lee SG, Song GW, Hwang S, Ha TY, Moon DB, Jung DH (2010). Surgical treatment of hilar cholangiocarcinoma in the new era:The Asan experience. J Hepatobiliary Pancreat Sci.

[3] Igami T, Nishio H, Ebata T, Yokoyama Y, Sugawara G, Nimura Y (2010). Surgical treatment of hilar cholangiocarcinoma in the “new era”:The Nagoya University experience. J Hepatobiliary Pancreat Sci.

[4] Kleive D, Berstad AE, Sahakyan MA, Verbeke CS, Naper C, Haugvik SP (2018). Portal vein reconstruction using primary anastomosis or venous interposition allograft in pancreatic surgery. J Vasc Surg Venous Lymphat Disord.

[5] Bacalbasa N, Balescu I, Vilcu M, Dima S, Iliescu L, Brezean I (2020). Superior mesenteric and portal vein reconstruction with cadaveric allograft during pancreatoduodenectomy - a case report and literature review. In Vivo.

[6] Nagino M (2019). Fifty-year history of biliary surgery. Ann Gastroenterol Surg.

[7] Labori KJ, Kleive D, Khan A, Farnes I, Fosby B, Line PD (2021). Graft type for superior mesenteric and portal vein reconstruction in pancreatic surgery - a systematic review. HPB (Oxford).

[8] Dokmak S, Aussilhou B, Sauvanet A, Nagarajan G, Farges O, Belghiti J (2015). Parietal peritoneum as an autologous substitute for venous reconstruction in hepatopancreatobiliary surgery. Ann Surg.

[9] Berumen J, Hemming A (2016). Vascular reconstruction in hepatic malignancy. Surg Clin North Am.

[10] Marino R, Tudisco A, Ratti F, Pedica F, Aldrighetti L (2023). Total portal vein replacement with peritoneal interposition graft during Whipple's procedure for extrahepatic cholangiocarcinoma:a technical report. World J Surg Oncol.

[11] De Pauw V, Pezzullo M, Bali MA, El Moussaoui I, Racu ML, D'haene N (2023). Peritoneal patch in vascular reconstruction during pancreaticoduodenectomy for pancreatic cancer:a single Centre experience. Acta Chir Belg.

[12] Felli E, Lapergola A, Pessaux P (2018). Autologous peritoneal patch for partial portal vein resection during left hemihepatectomy (with video). J Visc Surg.

[13] Radulova-Mauersberger O, Distler M, Riediger C, Weitz J, Welsch T, Kirchberg J (2022). How we do it - the use of peritoneal patches for reconstruction of vena cava inferior and portal vein in hepatopancreatobiliary surgery. Langenbecks Arch Surg.

[14] Sabuncuoglu MZ, Dandin O, Teomete U, Cakir T, Kayaalp C (2015). Using autologous peritoneal graft for portal vein injury due to blunt thoracoabdominal trauma. Hippokratia.

[15] Kumar NA, Bhandare MS, Chaudhari V, Sasi SP, Shrikhande SV (2019). Analysis of 50 cases of solid pseudopapillary tumor of pancreas:aggressive surgical resection provides excellent outcomes. Eur J Surg Oncol.

[16] Dokmak S, Aussilhou B, Marchese T, Kardoun N, Cauchy F, Schneck AS (2018). Right trisectionectomy and caval reconstruction with peritoneal patch under short total vascular exclusion for hepatocellular carcinoma with tumoral thrombus in suprahepatic vena cava. Ann Surg Oncol.

[17] Yekebas EF, Bogoevski D, Cataldegirmen G, Kunze C, Marx A, Vashist YK (2008). En bloc vascular resection for locally advanced pancreatic malignancies infiltrating major blood vessels:perioperative outcome and long-term survival in 136 patients. Ann Surg.

[18] Lapergola A, Felli E, Rebiere T, Mutter D, Pessaux P (2020). Autologous peritoneal graft for venous vascular reconstruction after tumor resection in abdominal surgery:a systematic review. Updates Surg.

[19] Elias D, Honoré C, Dumont F, Goéré D (2014). Autologous peritoneo-fascial graft:a technique for vascular reconstruction. J Visc Surg.

[20] Yoon SH, Yeo MK, Kim SH, Song IS, Jeon GS, Han SJ (2021). Feasibility of using the homologous parietal peritoneum as a vascular substitute for venous reconstruction during abdominal surgery:an animal model. Surgery.

[21] Har B, Balradja I, Krishna J, Agarwal S, Gupta S (2022). Parietal peritoneum as a vascular substitute for the reconstruction of donor inferior vena cava in living donor liver transplantation. J Liver Transplant.

